# Common Sense

**Published:** 1876-01

**Authors:** 


### COMMON SENSE.

NOT one in a multitude has it. Not one in a multitude of those who make use of the expression, knows what it means. Let the reader try this moment to define it in concise language, and in a moment he will find himself

"In endless mazes lost."

Yet it is a correct and appropriate phrase, if we can but distinguish between the possession and the exercise; the ownership and use of our senses. The word "common" qualifies as to the amount of sense, but does not apply to its use. The exact meaning to be attached to the expression is the *use* of an amount of intelligence which the mass of persons possess. *Common sense is the* USE *of experience and observation.* It is the practical employment of an ordinary amount of intelligence. Most persons have it—few use it. Its possession is common—its practice uncommon; hence the literal correctness of the expression—"*Very few people have common sense.*" It would be plainer to say—"Very few people *make use* of their common sense." For example—

Ask the first man you meet if he has not pushed up his wristbands, in washing his hands, with a view to their remaining up, to prevent wetting them, until the operation is over. Ask him, further, if he has not done the same thing a hundred times, and if, in a single instance, he ever knew them to stay up until he was done. And yet, that man, until the day of his death, will attempt that same useless thing, as often as he has occasion to wash his hands with his coat on, or without the trouble of unbuttoning the wristbands. He has, in common with the multitude, sense enough to know that the wristbands will not stay up, but yet he does not *use* his intelligence. Hence, it is appropriately said of that man, "He has not common sense"—that is, he does not *exercise* common sense.

A man knows how to be polite. He may be in a company which does not merit its exercise, in his opinion—still, the omission of it lays him liable to the charge, "He has no politeness"—that is, he does not practice it.

The mass of people know that jumping out of a vehicle, when the horses are running away, is very certain to be followed with loss of limb or life; they know, too, that dropping one's self out from behind is attended with comparatively little danger, and yet nine out of ten will jump out at the side—not one in a million will spill himself out from behind. Thus, every one of the million has sense enough to know the fact, yet, only one in a million is found to *use* it, to *practice* his knowledge.

Any body has sense enough to know, that, if additions are daily made to any vessel, and nothing be taken from it, day after day, the vessel will soon overflow, and there will be mischief and loss; and yet, there are multitudes in every community who ruin their health in early life, preparatory to a premature death, or an age of suffering, by eating heartily two or three times a day, for days together, without heeding the necessity of a daily action of the bowels, as a preventive of irretrievable mischief. Countless numbers of literary men, students, lawyers, clergymen, lose their health, and are laid aside from usefulness and duty, by failing to recognize practically a principle so self-evident, that daily additions to the contents of the body, without a proportionate outlet, must result disastrously. Thus it is, we say of many great men—men of extraordinary acquirements—all their talents cannot preserve them from poverty. They have the sense, but do not *use* it.

